# Nano-particle vaccination combined with TLR-7 and -9 ligands triggers memory and effector CD8^+^ T-cell responses in melanoma patients

**DOI:** 10.1002/eji.201142361

**Published:** 2012-07-18

**Authors:** Simone M Goldinger, Reinhard Dummer, Petra Baumgaertner, Daniela Mihic-Probst, Katrin Schwarz, Anya Hammann-Haenni, Joerg Willers, Christine Geldhof, John O Prior, Thomas M Kündig, Olivier Michielin, Martin F Bachmann, Daniel E Speiser

**Affiliations:** 1 Dermatology and Pathology Departments, University Hospital of ZurichZurich, Switzerland; 2 Clinical Tumor Biology & Immunotherapy Unit, Ludwig Center of the University of LausanneLausanne, Switzerland; 3 Nuclear Medicine Department, University Hospital CenterLausanne, Switzerland; 4 Cytos Biotechnology AGSchlieren-Zurich, Switzerland

**Keywords:** IFA (Montanide), Imiquimod, Melanoma, Melan-A/MART-1 antigen, Peptide-based vaccination

## Abstract

Optimal vaccine strategies must be identified for improving T-cell vaccination against infectious and malignant diseases. MelQbG10 is a virus-like nano-particle loaded with A-type CpG-oligonucleotides (CpG-ODN) and coupled to peptide_16–35_ derived from Melan-A/MART-1. In this phase IIa clinical study, four groups of stage III-IV melanoma patients were vaccinated with MelQbG10, given (i) with IFA (Montanide) s.c.; (ii) with IFA s.c. and topical Imiquimod; (iii) i.d. with topical Imiquimod; or (iv) as intralymph node injection. In total, 16/21 (76%) patients generated ex vivo detectable Melan-A/MART-1-specific T-cell responses. T-cell frequencies were significantly higher when IFA was used as adjuvant, resulting in detectable T-cell responses in all (11/11) patients, with predominant generation of effector-memory-phenotype cells. In turn, Imiquimod induced higher proportions of central-memory-phenotype cells and increased percentages of CD127^+^ (IL-7R) T cells. Direct injection of MelQbG10 into lymph nodes resulted in lower T-cell frequencies, associated with lower proportions of memory and effector-phenotype T cells. Swelling of vaccine site draining lymph nodes, and increased glucose uptake at PET/CT was observed in 13/15 (87%) of evaluable patients, reflecting vaccine triggered immune reactions in lymph nodes. We conclude that the simultaneous use of both Imiquimod and CpG-ODN induced combined memory and effector CD8^+^ T-cell responses.

## Introduction

Melanoma is associated with frequent spontaneous CD8^+^ T-cell responses that may improve the clinical outcome of the disease. Moreover, immunotherapy has demonstrated clinical benefit for melanoma patients, particularly after adoptive T-cell transfer, or treatment with anti-CTLA-4 antibodies 1–3. CD8^+^ cytotoxic T cells recognize a wide variety of tumor-associated antigens including melanocytic differentiation antigens, shared tumor-specific antigens, and mutated antigens, as listed in the T cell-defined tumor antigen database at http://cancerimmunity.org/peptide/. These antigens are presented as peptides by human leucocyte antigen (HLA) molecules on the surface of tumor cells and antigen presenting cells (APCs). The most potent inducers of T cells are the dendritic cells (DCs), which must be activated by innate stimuli and cytokines for optimal immunogenicity.

In contrast to passive immunization, active vaccination is thus far unable to demonstrate clinical benefit in most studies 4, 5. Improvement of cancer vaccines depends on continued investments in research and development, and a better understanding of innate and specific immune activation pathways 6, 7. Currently, cancer vaccines are still relatively inefficient in the generation of therapeutic T-cell responses; this is also the case for vaccines against infectious diseases, where efficient and protective memory and effector T-cell responses are rarely induced.

MelQbG10 is an innovative vaccine that integrates three components essential for successful immunotherapy. First, MelQbG10 consists of an immunogenic virus-like nano-particle (VLP), that is a protein shell with a diameter of 30 nm derived from the bacteriophage Qbeta that efficiently drains into local lymph nodes for uptake and processing by DCs and macrophages 8. Second, the VLPs contain short immunostimulatory oligonucleotides called G10, an unmodified A-type CpG-ODN triggering toll-like receptor (TLR)-9, protected from DNAse attack by the VLP. And third, the peptide_16–35_ of the antigen Melan-A/MART-1 is covalently coupled to the VLP. The peptide is derived from the melanocyte differentiation antigen Melan-A/MART-1, is part of the melanosome, and is expressed in early and advanced melanoma lesions 9. Upon injection, MelQbG10 is taken up by DCs, which mature and present Melan-A/MART-1 peptides on their surface and activate cytotoxic T lymphocytes (CTLs) 10, as well as Th cells 11. T-cell activ-ation is further promoted by the CpG-ODN G10 that triggers B cells and plasmocytoid DCs via TLR-9. This results in high-level expression of costimulatory molecules (CD80, CD86) and secretion of various cytokines (e.g. IFN-α, TNF-α, IL-12) supporting proliferation and maturation of immune cells.

In a first clinical trial, we showed that vaccination with MelQbG10 was well tolerated and can be used safely in melanoma patients 10. Proof-of-concept was provided by the induction of tumor-specific CD8^+^ T cells after MelQbG10 vaccination 10. Nevertheless, it is necessary to further improve immune responses and clinical responses. The aim of the present study was to further increase the immunogenicity of MelQbG10. Therefore, we used Montanide ISA-51 (Incomplete Freund's Adjuvant (IFA)), an oil-based depot-like adjuvant widely applied to boost immunogenicity of various antigens in many animal models and in clinical trials 12–14, and Imiquimod 5%, a cream that is known to activate APCs, including plasmacytoid DCs, via TLR-7 in the skin 15, 16 to determine whether the immunogenicity of MelQbG10 may be further enhanced by additional immune stimulatory agents.

## Results

### Safety and tolerability

**Figure 1:**
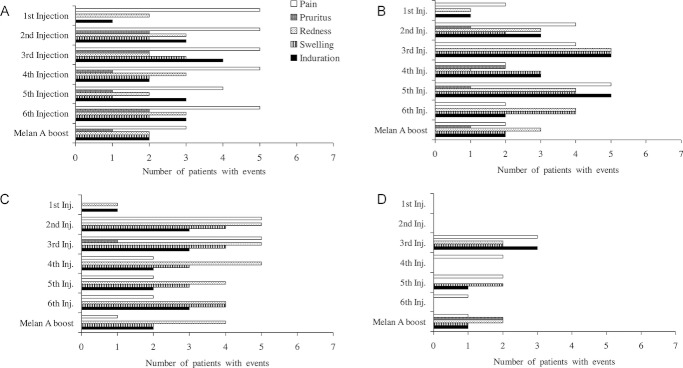
Incidence of injection site reactions per treatment group. The number of patients with pain and/or itching and the number of patients with a diameter ≥ 1 cm with regard to local erythema, swelling, and/or induration were enumerated in the available diary recordings. The results are shown for each injection. (A) Group I: local reactions after 1 mg s.c injection of MelQbG10 with IFA (*n* = 5) and (B) Group II: with IFA and topical Imiquimod 5% (*n* = 6); (C) Group III: i.d. injection with topical Imiquimod 5% (*n* = 5); and (D) Group IV: after ultrasound-guided intralymph node injection (14/42/140 μg MelQbG10, *n* = 5).

Twenty-one patients were enrolled, whereof 17 patients finished the clinical trial according to protocol and four patients discontinued earlier due to disease progression (one patient per group). Baseline characteristics of the patients are shown in Supporting Information Table 1. A total of 122 injections were applied according to the four treatment regimens. The vaccine was generally well tolerated with transient local reactions at the injection sites mostly rated as of mild intensity. The 21 patients experienced a total of 187 adverse events (AEs). Of those, 12 events emerged between screening and first vaccination. Sixty-two AEs (35%) were judged by the investigator as of causal relationship to the study drug. Of all AEs, 185 AEs were judged by severity, defined as mild, moderate or severe: 154 (83%) of the AEs were mild, 30 (16.5%) moderate and 1 (0.5%) severe. Six events were judged as serious (SAEs according to ICH guidelines) that were hospitalizations due to tumor progression. No SAE was considered to be related to the study treatment. Most recognized AEs were local adverse reactions (induration, pain, erythema, and swelling). Patients of treatment groups I and II (with IFA) showed more treatment related local AEs (40 versus 22 AEs) than patients of groups III and IV (without IFA). Injection site reactions were less frequent and milder after intranodal injection than after i.d. or s.c. administration (Fig. 1). Pain was predominant in patients receiving IFA, whereas erythema and swelling was predominant in Imiquimod-treated patients. Most local reactions were observed after the third injection in all treatment groups. Fatigue and flu-like symptoms were mainly found in IFA-treated patients (Table 1). Vital signs, ECGs, and laboratory parameters were all unremarkable pre- and posttreatment.

**Table 1:** All adverse events with an incidence > 3, listed for each group with number of events and number of patients

Preferred term	Group I events (patients)	Group II events (patients)	Group III events (patients)	Group IV events (patients)
General disorders and administration site conditions				
Injection site induration	4(2)	15(5)		1(1)
Influenza-like illness	2(2)	2(1)		7(2)
Fatigue	4(3)	2(2)		10(4)
Edema peripheral	4(2)			1(1)
Skin and subcutaneous tissue disorders				
Skin neoplasm excision	1(1)		8(3)	3(2)
Skin nodule	2(2)	5(3)	1(1)	3(2)
Blood and lymphatic system disorders				
Lymphadenopathya)	1(1)	1(1)	6(4)	4(3)

a) Defined as clinically palpable or radiologically enlarged lymph nodes.

### T-cell frequencies

**Figure 2:**
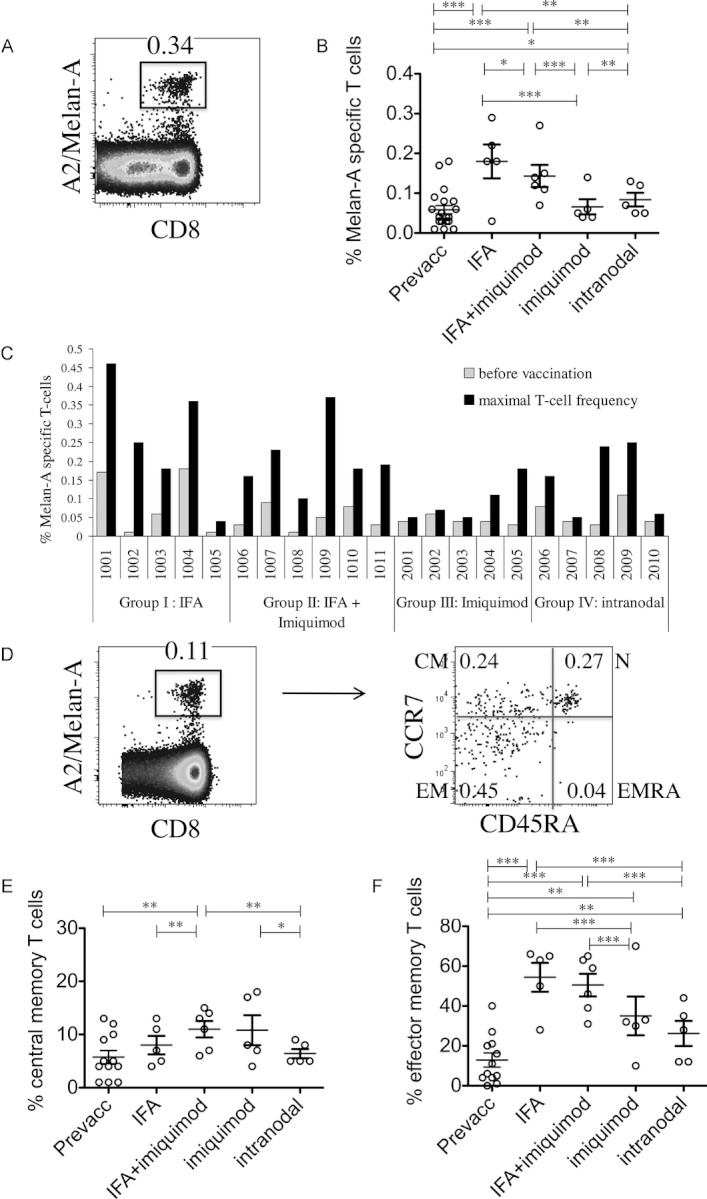
Frequency and memory-/effector-phenotype cell differentiation of Melan-A/MART-1-specific T cells. PBMCs were analyzed by flow cytometry directly ex vivo, that is without prior in vitro cultures. (A) Representative dot plot from one patient out of 21 (patient 1001-group I), showing staining for CD8 expression and Melan-A/HLA-A2 tetramers. (B) T-cell frequencies of the four patient groups, before (Prevacc) and after vaccination s.c. adjuvanted with IFA (group I, five patients), IFA with topical Imiquimod 5% (group II, six patients), i.d. injection with topical Imiquimod 5% (group III, five patients) or intranodal injection (group IV, five patients). (C) T-cell frequencies for each patient, before vaccination and the highest level reached after start of vaccination. (D) Representative dot plot from one patient of 21 (patient 1009, group II) of gating of Melan-A-specific T cells and staining with CCR7 and CD45RA-specific antibodies. (E) Percentages of central-memory (CM; CCR7^+^/CD45RA^−^) phenotype cells among Melan-A-specific T cells of the four treatment groups. (F) Percentages of effector-memory (EM; CCR7^−^/CD45RA^−^) phenotype cells (*n* = 5 for patient groups I, III, IV, and *n* = 6 for patient group II). (A and D) Values indicate percentages of CD8^+^ T cells and of Melan-A-specific T cells. (B, E, and F) Data shown are mean values per patient, calculated from the results obtained with blood samples from all studied six time points after vaccination, as detailed in the Supporting Information Fig. 1. ***p<0.001, **p = 0.001–0.01, and *p = 0.011–0.049, Mann–Whitney test.

The primary aim of the study was to activate Melan-A-specific CD8^+^ T cells. PBMCs were analyzed by flow cytometry with fluorescent peptide/HLA-A2 tetramers. In total, 16 of 21 patients (76%) were immune responders to vaccination with MelQbG10, as defined by at least twofold increased percentages of Melan-A tetramer positive cells after start of vaccination in comparison to before 17. T-cell frequencies were increased in all 11 patients of treatment groups I and II, that is after vaccination with MelQbG10 together with IFA, with or without local application of Imiquimod (Fig. 2A–C and Supporting Information Table 2). Two patients in treatment group III and three in treatment group IV also reached maximal T-cell frequencies fulfilling the T-cell responder criteria. Nevertheless, in patients vaccinated without IFA but treated topically with Imiquimod (group III) and by intranodal injection (group IV) the T-cell frequencies were significantly lower as compared to patients after MelQbG10 vaccinations with IFA (groups I and II, *p* = 0.003).

Patient age varied slightly from patient group I to IV (means of 58, 49, 61, and 56 years, respectively; Supporting Information Table 1). We cannot formally exclude that these differences biased our results. However, it seems rather unlikely, as there was no significant correlation; young patients did not differ significantly from older patients with regard to their T-cell frequencies (Supporting Information Table 2). This finding fits with our recent results from another study also showing that T-cell responses to CpG-based vaccination did not correlate with patient's age 18, compatible with a laboratory study demonstrating that CpG used as vaccine adjuvant can compensate for eventually reduced immune responsiveness in aged mice 19.

### Antibody responses

All 21 patients developed marked humoral immune responses showing serum Melan-A- and Qb-specific IgG antibodies in ELISA. Antibody titers in patients of groups I and II were statistically significantly higher than titers in patients of groups III and IV, in accordance to the cellular responses described above (Supporting Information Fig. 2).

### T-cell differentiation

**Figure 3:**
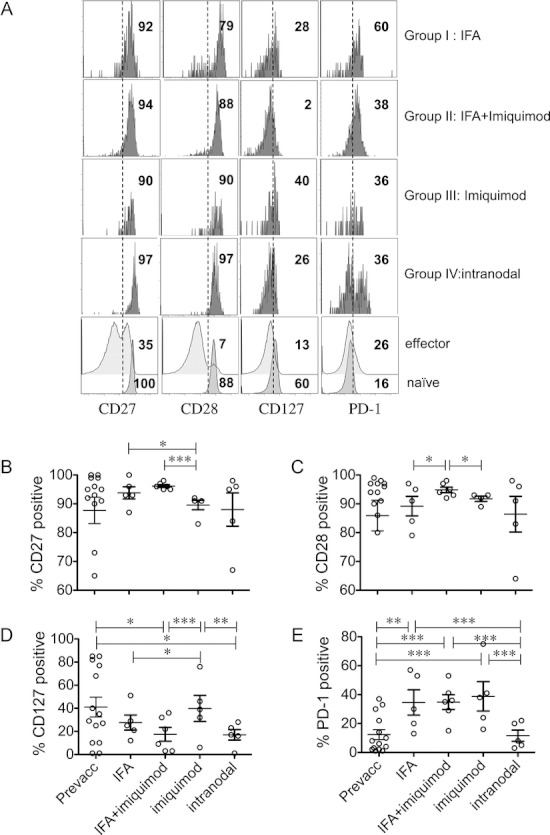
Expression of surface receptors by Melan-A/MART-1-specific T cells. Melan-A-specific T cells were gated similarly as in Figure 2, and analyzed for expression of the costimulatory molecules CD27 and CD28, the IL-7 receptor CD127 and the inhibitory receptor PD-1. (A) Representative dot plot from one patient out of *n* = 5–6 for each treatment group, that is patient 1001 (group I), patient 1009 (group II), patient 2002 (group III), and patient 2006 (group IV). As a reference, histograms in the lowest row show naïve (CCR7^+^/CD45RA^+^/tetramer^−^) and effector (CCR7^−^/CD45RA^+^/tetramer^−^) phenotype cells, that is subsets of “antigen-nonspecific” CD8^+^ T cells from the representative patient 1009. Values in histograms indicate percent positive cells. (B–E) Statistical comparisons between the four treatment groups for expression of (B) CD27, (C) CD28, (D) CD127, and (E) PD-1 by Melan-A-specific T cells. The data shown in panel B–E are mean values per patient, calculated from the results obtained from all studied time points after vaccination, similarly as for Figure 2. *** <0.001, ** 0.001–0.01, and * 0.011–0.049, Mann–Whitney test.

The proportions of Melan-A-specific T cells at the various differentiation stages were determined by staining with tetramers, combined with antibodies specific for the short isoform of CD45 (CD45RA) and the chemokine receptor CCR7 20. Double negative T cells, so-called effector-memory (EM)-phenotype cells, were significantly increased after IFA adjuvanted vaccination (Fig. 2F). In contrast, the proportions of central-memory (CM)-phenotype cells (CCR7^+^ CD45RA^−^) were increased in patient groups II and III who had been treated topically with Imiquimod (Fig. 2E). Multiparameter flow cytometry allowed simultaneous analysis of four additional receptors. The two costimulatory molecules CD27 and CD28 are known to be progressively downregulated in CD8^+^ effector T cells. There was a trend to lower expression of CD27 and CD28 after vaccination adjuvanted with only IFA or only Imiquimod, but the differences to before vaccination were not significant (Fig. 3A–C). In accordance with the highest proportions of CM-phenotype cells, Imiquimod was also associated with the highest frequencies of CD127^+^ (IL-7R) cells, which was significantly higher in comparison to each of the other three patient groups (Fig. 3D). Finally, we also determined PD-1 expression by the Melan-A-specific T cells. Interestingly, the mean values in the first three patient groups were all approximately 32–34% and thus significantly higher than before vaccination and higher than in patient group IV with a mean value of only 14% (Fig. 3E). Figures 2 and 3 show the patient's mean values from all time points after vaccination. Example data from a single time point (after six vaccinations with MelQbG10) are provided in Supporting Information Fig. 3, showing similar differences between the four patient groups. However, in many instances the differences did not reach statistical significance, likely due to the low numbers of samples.

### Clinical and PET/CT imaging results

Taking into account all medical and laboratory data acquired during the trial, the disease status could be evaluated for 14 of the 21 patients. Not all target lesions could be followed throughout the 9 month study period due to surgical excisions and due to different imaging schedules determined by the investigators. Disease progression according to the investigator's judgment was documented in nine patients: group I 2/5, group II 2/6, group III 3/5, and group IV 2/5 (see also Supporting Information Table 2). Stable disease was documented in five patients: group III 2/5 and group IV 3/5. A follow up evaluation of the disease status was obtained in April 2010 for all 21 patients. In group I, three patients showed progressive disease (PD), whereas two patients remained without evidence of disease. In group II, two patients showed PD whereas four patients had no evidence of disease. In both groups III and IV, two patients had died by the time of the follow up, whereas three patients had no evidence of disease.

**Figure 4:**
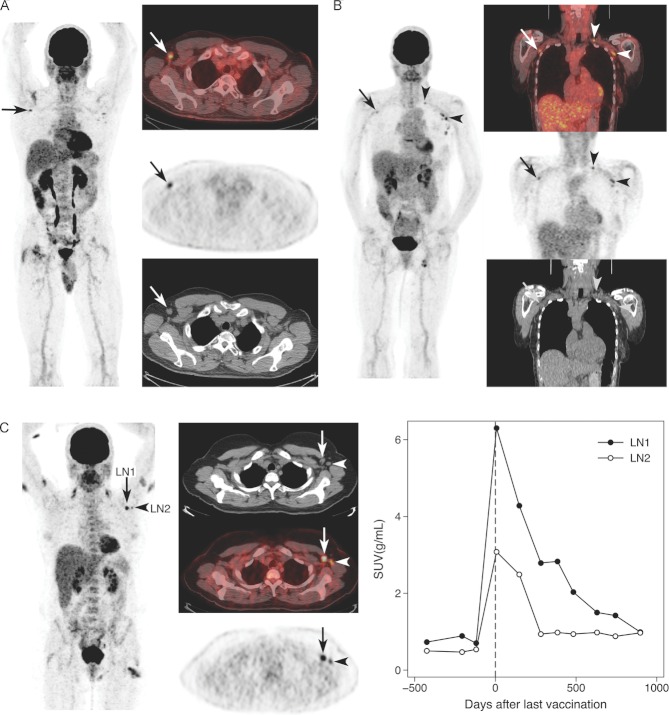
PET/CT imaging showing enlarged lymph nodes and increased glucose metabolism after vaccination. Fifteen of the 21 patients of this study underwent PET/CT imaging. In parallel to the assessment of disease development (not shown), immune activation was evaluated by measuring the glucose metabolism in vaccine-site draining lymph nodes. (A) Patient 2002 (group III) with bilateral axillary lymph nodes with one that is enlarged (arrow). Images: Maximal intensity projection (left) with PET/CT fusion image (top), PET image (middle), and CT image (bottom). (B) Patient 2004 (group III) with multiple bilateral axillary (arrow, horizontal arrowhead) and subclavicular (vertical arrowhead) lymph nodes with increased glucose metabolism. (C) Patient 1003 (group I) with long-term longitudinal follow-up before and after the seven vaccinations injected between the third and fourth PET/CT studies. The standardized uptake value (SUV) was measured in two axillary lymph nodes (LN1, LN2) from patient 1003 (group I) before and after the last vaccination (right). Note also the local inflammation in vaccination sites in the proximal right and left arms and left thigh (left).

Independently from disease status, lymphadenopathy defined as clinically palpable or radiologically enlarged lymph nodes was observed in nine patients in average several weeks after study entry (Supporting Information Table 2). In seven patients, this lymphadenopathy involved all palpable lymph node regions. In these seven patients PET/CT-scans showed lymph node enlargement and increased glucose up-take. In order to exclude metastatic nodes, fine needle aspirates, and regular clinical follow-up including additional imaging and serum S-100 were performed showing no melanoma metastatic cells. Furthermore, these activated and enlarged lymph nodes were observed in patients without evidence of disease, and/or in body regions that were not affected by melanoma. Interestingly, in 13 of 15 patients (87%) who underwent PET/CT imaging, a significantly increased metabolic activity was seen (SUV = 3.5 ± 1.5 versus 0.92 ± 0.02 g/mL, *p* = 0.01), predominantly localized in the upper torso in enlarged lymph nodes (Fig. 4A and B). There was no observed significant difference in glucose metabolic activity among study groups (*p* = 0.17), although the study might have been underpowered to detect one. Most observed lymph node metabolic activity were seen in axillary lymph nodes (10/12 or 83%), often bilaterally and in 2–11 (median 2, interquartile range 2–7) separate lymph nodes; less often increased activity was seen in inguinal or external iliac lymph nodes (4/12 or 33%). We tested if an association existed between maximally reached percentages of Melan-A-specific T cells as well as with the value at PET/CT and the glucose metabolism reflected by PET/CT SUV, but this could not be confirmed (p > 0.7). In one patient, extensive imaging follow-up was available by PET/CT with three examinations before the first vaccination and eight after the full course of vaccination (Fig. 4C). Significantly increased uptake in PET/CT-measured metabolism was seen bilaterally in axillary lymph nodes with the two largest increases in the left axilla (SUV = 6.3 and 3.1 g/mL; Fig. 4C). Over a period of 870 days, metabolic activity returned to baseline, with differences still visible at 716 days after the last vaccine injection in the largest lymph node and at 120 days in the smaller one (Fig. 4C).

### Tumor tissue from patients with relapse

**Figure 5:**
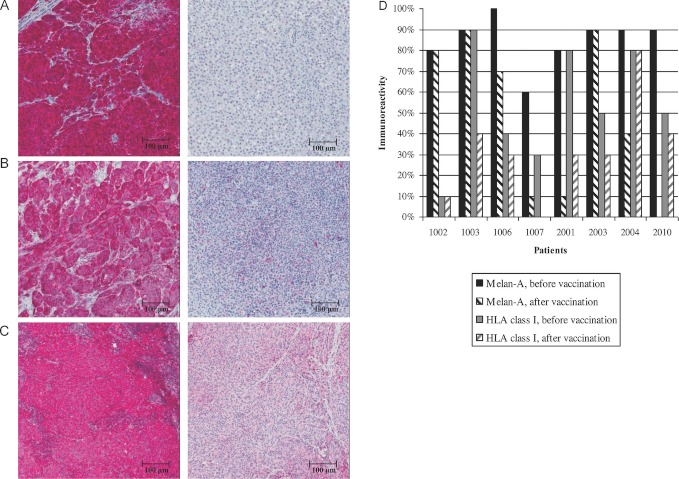
Reduced HLA-class I and Melan-A/MART-1 expression after vaccination. Immunohistochemistry images (×8 magnification) of different tumor biopsies before (left) and after (right) vaccination showing reduced Melan-A or HLA-class I expression. (A) Tumor biopsies from plantar left (primary tumor, left) and left leg (in transit cutaneous metastasis, right) with high (left) and low (right) Melan-A intensity staining (Patient 2010, group IV). (B) Biopsies of two dermal abdominal metastases, with high (left) and low (right) Melan-A intensity staining (Patient 2001, group III). (C) Cutaneous metastasis of the right knee (right and left) with low HLA-class I intensity staining (Patient 1003, group I). (D) Melan-A and HLA-class I expression before and after vaccination, for each evaluable patient (*n* = 8) are shown.

In 8 patients tumor tissue from skin and subcutaneous lesions was collected prior and after vaccination. We analyzed the expression of Melan-A and HLA-class I (Fig. 5 and Supporting Information Table 2). Diminished expression of Melan-A or HLA-class I was defined by more than 50% reduced staining of intensity. This was the case for Melan-A in 4/8 patients (one patient in group II, two patients in group III and one patient in group IV) and for HLA-class I in 2/8 patients analyzed (one patient in group I and one patient in group III; Fig. 5D).

## Discussion

In this trial, we analyzed the T-cell induction properties and the safety profile of MelQbG10 when combined with different adjuvants (IFA, Imiquimod 5%) and/or administered by different routes (s.c., i.d., and i.n.). The vaccinations were well tolerated; there were no major toxicities. We did not observe autoimmune-related reactions, in contrast to the recently introduced immunotherapy with anti-CTLA-4 antibody (Ipilimumab) 21. As expected, there were in part intense and persistent local reactions, particularly in patients who received IFA 22, 23. Furthermore, we observed (multi-) regional lymphadenopathy. Interestingly, this was associated with in part high up-take of glucose detected in PET/CT scans, mimicking metastatic involvement. This phenomenon can also be termed pseudo-progression. It reflects a significant activation of vaccine site draining lymph nodes. Similar reactions have already been observed with IFN-α treatment 24 and with Ipilimumab.

VLPs are very efficient in eliciting antibody responses, and thus frequently used for various vaccine purposes 25. Therefore, it was not surprising that anti-Melan-A antibodies were induced efficiently. It is likely that the observed anti-Melan-A antibodies are directed to several B-cell epitopes of the long Melan-A peptide used for vaccination. Possibly, some of these epitopes may overlap with the sequence of amino acids 26–35 representing the A2/Melan-A T-cell epitope. Besides antibodies and CD4 T-cell responses 11, VLPs can also trigger CD8^+^ T-cell responses. It is generally acknowledged that clinically useful T-cell responses are much more difficult to generate as compared to antibody responses. Therefore, T-cell vaccines are much less advanced as opposed to B-cell vaccines. Numerous academic and industrial teams undertake multiple research and development efforts, in order to elucidate the precise biological parameters that need to be considered for generating T-cell responses that are capable to protect from infectious or malignant disease. In this mindset, we are optimizing VLP-based vaccines.

In a previous study in melanoma patients, we have shown that MelQbG10 can trigger ex vivo detectable and thus relatively strong tumor-specific CD8^+^ T-cell responses 10. The present study was based on the same vaccine (MelQbG10), but extended by additional components. The addition of IFA resulted in significantly increased T-cell frequencies. These responses were dominated by EM-phenotype cells, that is CD45RA^−^CCR7^−^ cells. These cells were also mostly CD127 (IL-7R) negative, demonstrating that IFA primarily induced effector-phenotype cells, with only low proportions of memory-phenotype cells. These results are reminiscent of vaccines composed of synthetic peptide, CpG-ODN, and IFA, which are also dominated by EM-phenotype CD8^+^ T cells 26. In contrast, our data show that increased percentages of CM-phenotype cells (CD45RA^−^CCR7^+^) with enhanced expression of CD127 were induced by the addition of Imiquimod. Thus, triggering TLR-7 by Imiquimod may promote memory differentiation, which is promising because memory cells are known to increase the protective potential of T-cell responses 27–29. Despite its importance, memory cell triggering is often insufficient by synthetic vaccines. The mechanisms by which TLR-7 may promote memory cell activation are likely linked to DC activation via TLR-7. The generation of memory cells may depend on Wnt-TCF-1, mTor, and AMPK signaling pathways 27–29. Our data point to possible connections between these mechanisms and TLR-7 induced pathways.

Our previous study showed that even without topical Imiquimod vaccination with MelQbG10 induced increased percentages of CM-phenotype cells 10. Here, we show that the combination of MelQbG10 plus Imiquimod and IFA (thus triggering of TLR-7 and -9) leads to enhance promotion of memory-phenotype cells. However, we have never tested whether this can be achieved by triggering of TLR-7 without TLR-9, since MelQbG10 always contained the CpG-ODN G10.

Recent literature reported that the combined stimulation of multiple innate immune receptors may enhance vaccine efficacy 30, reminiscent to the notion that pathogens may trigger innate immunity due to the expression of multiple microbe-associated molecular patterns (MAMPs). However, there are still considerable limitations in the availability of clinically graded drugs for triggering innate immune receptors. Imiquimod is a skin cream and can only be applied topically. It will be interesting to test whether systemic and thus more efficient TLR-7 triggering can promote even higher numbers of CM-phenotype cells.

The frequencies of Melan-A-specific T cells after vaccination with MelQbG10 plus Imiquimod were relatively low. In contrast, the addition of IFA resulted in higher frequencies. Thus, combining CpG-ODN, Imiquimod, and IFA achieved both memory- and effector-phenotype cells, with relatively high frequencies and high proportions of CM-phenotype cells. However, our data are pre-liminary due to the low numbers of patients, and thus require further confirmation.

Even though the percentages of tetramer positive T cells were relatively low, they actually revealed large numbers of tumor-specific T cells, because we analyzed the blood samples directly ex vivo. In many cancer vaccine studies, T cells are analyzed after they have proliferated in tissue cultures for one or several weeks, providing results with higher T-cell percentages but precluding precise conclusions on T-cell numbers and qualities in vivo 12. Therefore, recent cancer vaccine studies increasingly include analysis of T cells directly ex vivo 31–38. Because of considerable inter-patient variability of frequencies of tumor-antigen-specific T cells, at baseline and after immunotherapy, it is important to determine how many patients are “immune–responders”, based on the comparison of their values before versus after vaccination. Data from direct ex vivo analysis show that the majority of vaccination strategies were able to trigger responses of Melan-A-specific CD8^+^ T cells but usually not in all patients 31–38. For example, a recent study by Ribas et al. showed T-cell responses in about 50% of melanoma patients vaccinated with DNA followed by peptides, a novel prime-boost approach 36. The results of our present study with 16/21 immune responder patients is favorable, particularly when focusing on the 11 patients in whom vaccination included IFA (groups I and II) who all showed ex vivo detectable T-cell responses. However, the T-cell frequencies remained lower than in patients vaccinated with peptide + CpG 7909 (PF-3512676) emulsified in IFA 18, 26, raising the possibility that B-type CpGs (e.g. 7909) may be superior to A-type CpGs (e.g. G10) for CD8^+^ T-cell vaccination in humans. Definitive conclusions depend on trials directly comparing the various vaccination approaches. Importantly, clinical efficacy needs to be tested in large-scale trials. The currently available evidence of clinical benefit of CpG-based vaccines 18 justify evaluating their clinical usefulness in phase III studies.

We developed MelQbG10 several years ago, and used the analog peptide sequence ELAGIGILTV with L instead of A at position 2 as compared with the natural sequence EAAGIGILTV. As for other analog peptides, we found that this analog peptide triggered relatively high frequencies of T cells. However, more recently we found that T-cell responses were more robust after vaccination with the natural peptide, since the T cells were more strongly activated and were of higher functional avidity as compared with T cells after vaccination with the analog peptide 39. Therefore, it is preferable to vaccinate with peptides corresponding to native tumor antigens. Unfortunately, such peptides are often insufficiently antigenic, particularly when cross-presentation is required such as for long peptides as used in this study. Therefore, immunization with analog peptides is still justified since reasonably large fractions (>50%) of the induced T cells can recognize tumor cells 39. The question whether the native or the analog Melan-A peptide should be used for T-cell analysis is much easier to answer. Tetramers constructed with the Melan-A analog peptide are “universal,” as they readily bind virtually all T cells induced by analog or natural peptide, but also endogenously by the tumor itself 39. Thus, A2/Melan-A tetramer binding is highly cross-specific and does not discriminate between the very fine differences in specificity and affinity depending on vaccination with analog versus native antigen.

Of interest, PET/CT could evidence increased lymph node activity in the majority of the patients, as a way of showing increased activity in relation to inflammation and immune response, even in the long term (>4 months). This has not been utilized in vaccine studies so far. It has rather been described as an epiphenomenon in relation to the last vaccination campaigns against influenza A/H1N1 pandemic as seen in patients undergoing oncologic PET/CT 40–42 or in sporadic immunization cases 43, 44. Increased glucose metabolism in lymph nodes was not observable >14 days after immunization in the study by Burger et al. 42 or >30–50 days by Thomassen et al. 41, possibly due to the shorter stimulation time of these vaccines as opposed to the vaccine formulations used in the present study. Interestingly, Iyengar et al. 45 found an association between PET-measured lymph node signals and viremia in HIV-infected patients that was attributed to CD4 lymphocyte activation in relation to HIV replication. They observed a predominance for upper torso lymph nodes in recently and chronically HIV-infected patients, but not in their control group of healthy non-HIV-infected volunteers undergoing killed influenza vaccination, where it was observed only on the upper torso of the injection site. In future trials, PET/CT could be an attractive way to noninvasively monitor and investigate inflammatory and immune responses to vaccination, although the exact mechanisms and significance of the increased glucose metabolism remain to be investigated.

In summary, MelQbG10 can be considered safe and well tolerated when given at a cumulative dose of 6 mg (6 × 1 mg). The use of IFA resulted in more frequent side effects, but induced the highest Melan-A-specific T-cell frequencies. Both memory- and effector-phenotype T-cell responses were observed when MelQbG10/IFA vaccines were given in combination with Imiquimod. Our data suggest that the induced immune activation was biologically relevant because several relapsing melanoma lesions demonstrated diminished expression of Melan-A or of HLA-class I, presumable reflecting mechanisms of immune escape 46. Future vaccines need to target multiple antigens and HLAs simultaneously.

Vaccine components need to be tested in humans, in order to determine which of them, and what type of final vaccine formulations are most promising for further and large scale clinical development. We used the A-type CpG-ODN G10 that we had integrated in our VLPs, resulting in sizable T-cell responses, which were however less powerful than in rodents 10. In the present paper, we combined this approach with additional adjuvants and/or varied the administration route and found quantitatively and qualitatively different T-cell responses. The next steps will be to replace some components, by using, for example a B-type CpG-ODN, and/or by supplementation with (parenteral) drugs that trigger additional TLRs and further innate immune receptors. With a step-by-step approach, we will learn which type of vaccine formulation is optimal for triggering CTL and Th1 responses with the potential of clinical benefit for cancer patients.

## Materials and methods

### Patients and trial design

Eligible patients were at least 18-years old, with histologically confirmed stage III or IV malignant melanoma (AJCC), HLA-A*0201 positivity, and an expected survival of at least 9 months. The current general health condition had to allow the patient to undergo all study procedures according to the protocol. Main exclusion criteria were known or planned pregnancy or lactation, use of any investigational drug, and previous participation in a clinical trial with a Qb-based vaccine. The first patient was enrolled in May 2008, the last in December 2009. All patients provided written informed consent before any study-specific procedure was performed.

The exact composition of MelQbG10 was reported earlier 10. The first treatment group (group I) received three initial subcutaneous (s.c.) injections of 1 mg MelQbG10 mixed with IFA (Montanide ISA-51, Seppic GmbH, Koeln, Germany) in weekly intervals, followed by three monthly injections with the vaccine. The second treatment group (group II) was vaccinated identically to the first group but was additionally treated with topical Imiquimod 5% cream (Aldara™, 3M, Switzerland) once daily for 10 days (with 2 days of treatment pause after the first 5 days). The cream was applied with a thin layer on the injection site and covered an area of 5 × 5 cm. The third group (group III) received three intradermal (i.d.) injections of 1 mg MelQbG10 in weekly intervals, followed by three monthly intradermal injections with MelQbG10. In addition, the patients were treated with a thin layer of Imiquimod 5% cream once daily as described for group II. The fourth treatment group (group IV) received three ultrasound-guided intranodal (i.n.) injections of MelQbG10 initially starting with 14 μg, then 42 μg, and thereafter 140 μg in weekly intervals, followed by three monthly injections of 140 μg MelQbG10. All treatment groups received a final boost 4 weeks after the last vaccination with the Melan-A/MART-1 peptide plus IFA s.c (for more details on trial design see Supporting Information Fig. 1). Before starting treatment group II (Imiquimod + IFA) an internal safety data review was performed with respect to treatment group III (i.d. injection + Imiquimod). Local ethic review committees and responsible health authorities approved the study, which was performed according to GCP guidelines and registered at http://www.ClinicalTrials.gov: NCT00651703.

### Assessment of safety and tolerability and clinical results

Adverse events (AEs), concomitant medication, vital signs, physical examinations, ECGs, antinuclear antibodies, and routine clinical laboratory blood and urine analyses were monitored carefully at defined visits. Local reactions at the injection sites were recorded by patients and investigators. Patients were asked to record local reactions after every injection of study medication in a paper-based diary for a period of 3 days. Pain and itching were documented; swelling, and induration were assessed by recording the reactions’ diameter if the diameter was > 1 cm. Local reactions that required medical intervention were documented as AE. Safety and tolerability parameters were primarily evaluated within the single subject and compared with baseline, in addition, trends between the four groups were analyzed. In total 21 melanoma patients have been included and represented the safety population, which was identical to the full analysis set. No data have been extrapolated.

The status of the disease was monitored clinically and/or by computer tomography (CT) at screening and at the end of the trial, if clinically indicated a fluorodeoxyglucose-positron emission tomography (FDG-PET/CT) was performed (GE Healthcare, Milwaukee, WI, USA). To compare PET/CT imaging results, we used the maximal standardized uptake value (SUV in g/mL) corrected for body weight as a way to reflect glucose consumption.

### Immune monitoring and immunohistochemistry

Primary goal of the clinical trial was to achieve efficient Melan-A-specific T-cell responses by enhancing the immuno-stimulating potential of MelQbG10 with various adjuvants and different administration modes in patients with malignant melanoma. Simultaneous enumeration and phenotyping of antigen-specific CD8^+^ T cells was achieved by flow cytometry and were analyzed with FlowJo™ software (TreeStar). Ficoll-Paque centrifuged PBMC (1–2 × 10^7^) were cryopreserved in RPMI 1640, 40% FCS, and 10% DMSO. Phycoerythrin-labeled HLA-A*0201/Melan-A/MART-1 A27L peptide_26–35_ (ELAGIGILTV) tetramers were prepared as described 17. Anti-CD8, -CD28, -PD-1, and anti-CCR7 mAbs were purchased from BD PharMingen (San Diego, CA, USA), anti-CD45RA from Beckman Coulter (Brea, CA, USA), and anti-CD27 and -CD127 from eBioscience (San Diego, CA, USA). All tetramer and antibody batches were titrated to determine optimal reagent concentrations. PBMC were thawed, and CD8^+^ T cells were enriched using a MiniMACS device (Miltenyi Biotec, Bergisch Gladbach, Germany) resulting in >90% CD3^pos^ CD8^pos^ cells. Cells (10^6^) were incubated with tetramers (1 μg/mL, 60 min, 4°C) and then with antibodies (30 min, 4°C). For dead cell exclusion, cells were stained with Live/Dead Fixable Dead Cell violet stain (Molecular Probes/Invitrogen). A total of 5 × 10^5^ CD8^+^ T cells/sample were acquired with a flow cytometry LSR II™ machine. The cytometer performance was checked daily using the CST (BD™ Cytometer Setup and Tracking) quality control beads system according to the manufacturers’ instructions. To evaluate whether the CD8 T cells from the 21 patients of this study were representative for larger numbers of patients, we compared their pre-vaccination values to those of 37 untreated patients, also with stage III–IV metastatic skin melanoma, which we have recently studied with an identical flow cytometry approach. For all parameters studied (Melan-A tetramer, CD27, CD28, CD127, PD-1, and effector-memory-phenotype and central-memory-phenotype cells) there were no statistical differences (data not shown). Results of tetramer + T cells were calculated and are indicated in percentages of circulating CD8^+^ T cells. Results from T-cell phenotyping were only used when the numbers of tetramer positive events were at least 20.

Biopsies of metastases were stained for the melanocytic differentiation antigen and HLA-class I before and after the treatment. For immunohistochemistry the paraffin-embedded tumor tissue sections obtained at different time points prior and after vaccination were stained with anti-Melan-A/MART-1 monoclonal antibody A103 (Novocastra Laboratories Ltd., Newcastle upon Tyne, UK) 9 and with anti-HLA-class I antibody 3F10 (1:1000; RDI Research Diagnostics, Inc., Concord, MA, USA) 47. The quantification was done by an experienced, board-certified dermatopathologist (RD) on a Zeiss Axiophot HAL100 (Carl Zeiss Microimaging GmbH, Switzerland) with ×40 magnification. Immunoreactivity, evaluated as intensity of the staining in relationship to normal melanocytes in the epidermis (internal control), was scored semiquantitatively in percentages (0–100%). A clear cut difference, interpreted as diminished expression of Melan-A or HLA-class I, was considered starting from a change of 50% or more in the comparison of the sections prior to and after vaccination, as illustrated in Figure 5. Because of expression heterogeneity 9, we analyzed three high power fields of all lesions.

### Data and statistical analysis

Evaluation of the disease status was described for each patient individually. AEs were coded using MedDRA Version 12.0. Summary tables were presented by frequency of AEs and by patient. AEs were listed by subject, including verbatim term, coded MedDRA term, severity, and relationship to treatment. Standard laboratory measurements, vital signs, and ECG parameters were listed by subject and time point of collection. Out of normal range and clinically significant deviating values were summarized by descriptive statistics. Anti-Melan-A-specific T cells were expressed as % tetramer positive cells of total CD8^+^ T cells. Immune responders were defined as patients whose maximal T-cell frequency at any time point during the trial was at least double the preimmune T-cell frequency. T-cell frequencies below 0.01% were considered as not detectable (threshold) 17. Statistical significance was assessed by the nonparametric Mann–Whitney and Kruskal–Wallis tests or the parametric Student's *t*-test where appropriate. Associations were tested using nonparametric Spearman rank correlation. *P* values less than 0.05 were considered significant and reported in the figures. The scatter dot plots in Figure 2 and 3 show individual values, mean values, and standard deviations.

## Abbreviations

CT: computer tomography
FDG-PET: fluorodeoxyglucose-positron emission tomography
IFA: Incomplete Freund's Adjuvant
ODN: oligonucleotide
(S)AE: (serious) adverse event
VLP: virus-like nano-particle

## Supplementary material

Disclaimer: Supplementary materials have been peer-reviewed but not copyedited.

Supporting Information Table 1. Baseline Characteristics of Patients

Supporting Information Table 2. Overview of Results

Supporting Information Figure 1 Trial Design. Four groups of patients received vaccinations as indicated on the right side of the figure. For each patient, the total trial duration was approximately 9 months. The screening period took up to 4 weeks. The treatment phase, including the seven injections with MelQbG10, lasted 18 weeks and the follow-up phase ended after 36 weeks. All s.c. and i.d. injections were performed in the upper arm left or right alternatively. Injections with IFA were prepared by mixing equal volumes of MelQbG10 with IFA until the two liquids formed a homogenous emulsion. The intranodal injections were performed always in the same inguinal lymph node region. The lymph node dissected region was omitted for vaccine injections. No immunosuppressive medication was used during the study.

Supporting Information Figure 2 Anti-Melan-A and anti-Qb IgG Antibody responses. Geometric mean titers are expressed as reciprocal serum dilutions giving half-optical density in ELISA. Before vaccination (Visit 1) and at study end (Visit 12) anti-Melan A ( ♦) and anti-Qb (◊) titers were measured in patients’ blood serum samples. Panel A shows the titers of group I (1mg MelQbG10 + IFA s.c.), Panel B of group II (1mg MelQbG10 + IFA s.c. + Imiquimod 5%), Panel C of group III (1mg MelQbG10 i.d.+ Imiquimod 5%), and Panel D of group IV (14/42/140mg MelQbG10 i.n.). Mann-Whitney pair wise comparisons were performed. The contrasts of Group I and II vs. Group III and IV were statistically significant for anti-Melan-A and anti-Qb titers (p=0.003 and p=0.001, respectively).

Supporting Information Figure 3 Frequency and memory-/effector-phenotypes of Melan-A specific T cells. Results before vaccination (Prevacc) and of a single time point after vaccination with MelQbG10 (six vaccinations), from the same experiments as those for Figures 2 and 3, shown in a similar fashion. Statistical significances have been determined by the Mann-Whitney test. Where significant, P-values are symbolized with ** or * for < 0.01 or 0.01–0.049, respectively.
