# Supplementary material for: Nano-particle vaccination combined with TLR-7 and -9 ligands triggers memory and effector CD8+ T-cell responses in melanoma patients
Source: Eur J Immunol. 2012 Jul 18;42(11):3049–61. doi: 10.1002/eji.201142361 (PMC3549564; doi:10.1002/eji.201142361)
Supplement: Supplementary file 2 [file eji0042-3049-SD2.pdf]

Supporting Information Table 1. **Baseline Characteristics of Patients**

	Patient	Gender	Age [yrs]	Height[a)] [cm]	Weight [a)] [kg]	Melanoma Stage[a)]
**Group I: IFA**	1001	male	82	170	72.0	III
	1002	male	44	169	64.6	IV
	1003	female	62	158	72.0	III
	1004	female	29	185	68.0	III
	1005	male	72	185	65.0	III
**Group II: IFA**	1006	male	46	180	75.0	III
	1007	female	47	157	79.0	III
	1008	male	64	170	79.0	III
	1009	male	58	181	86.0	III
	1010	male	26	172	61.2	IV
	1011	male	53	171	75.0	III
**Group III: Imiquimod**	2001	male	61	175	104.0	III
	2002	male	61	175	92.8	III
	2003	male	56	174	89.2	III
	2004	female	69	163	58.2	III
	2005	female	56	168	55.0	III
**Group IV: intranodal**	2006	female	61	164	77.7	III
	2007	female	44	164	77.4	III
	2008	male	55	186	103.1	III
	2009	female	63	161	73.2	III
	2010	male	57	174	80.1	III

[a)] Assesed at screening.

Mean (± SD) patient age for patient groups I, II, III and IV was 58 ± 19, 49 ± 12, 61 ± 5 and 56 ± 7 years, respectively.

Supporting Information Table 2. **Overview of Results**

	Patient	Melan-A specific T cells [%]		Anti-Melan-A antibodies [ELISA titer]		Anti-Qb antibodies [ELISA titer]		Melan-A expression [%]		HLA class I expression [%]		Disease status at study end	Lymphadenopathy [b)]
		Before vaccination	Highest level after start of vaccination	Visit 1	Visit 12	Visit 1	Visit 12	Before vaccination	After vaccination	Before vaccination	After vaccination		
**Group I: IFA**	1001	0.17	0.46[a)]	281	26'470	< 1'000	1'050'000	-	-	-	-	-	
	1002	0.01	0.25[a)]	21	-	< 1'000	-	80	80	10	10	PD	√
	1003	0.06	0.18[a)]	10	10'627	< 1'000	697'845	90	90	90	40	PD	
	1004	0.18	0.36[a)]	57	43'977	< 1'000	1'370'000	-	-	-	-	-	
	1005	0.01	0.04[a)]	14	5'742	< 1'000	174'107	-	-	-	-	-	
**Group II: IFA +Imiquimod**	1006	0.03	0.16[a)]	25	16'511	< 1'000	452'528	100	70	40	30	-	
	1007	0.09	0.23[a)]	96	44'780	< 1'000	1'630'000	60	10	30	0	-	√
	1008	0.01	0.10[a)]	20	5'486	< 1'000	533'984	-	-	-	-	-	
	1009	0.05	0.37[a)]	52	-	< 1'000	-	-	-	-	-	PD	
	1010	0.08	0.18[a)]	225	23'531	< 1'000	610'473	-	-	-	-	PD	
	1011	0.03	0.19[a)]	28	8'245	< 1'000	476'915	-	-	-	-	-	
**Group III: Imiquimod**	2001	0.04	0.05	9	2'273	< 1'000	228'223	80	10	80	30	PD	√
	2002	0.06	0.07	-	1'097	-	14'852	-	-	-	-	SD	√
	2003	0.04	0.05	8	-	< 1'000	-	90	90	50	30	PD	
	2004	0.04	0.11[a)]	116	9'587	< 1'000	204'271	90	40	80	80	SD	√
	2005	0.03	0.18[a)]	30	-	< 1'000	-	-	-	-	-	PD	√
**Group IV: intranodal**	2006	0.08	0.16[a)]	36	736	< 1'000	23'520	-	-	-	-	SD	
	2007	0.04	0.05	26	278	< 1'000	5'817	-	-	-	-	PD	
	2008	0.03	0.24[a)]	-	6'216	-	35'725	-	-	-	-	SD	√
	2009	0.11	0.25[a)]	12	1'827	< 1'000	34'598	-	-	-	-	SD	√
	2010	0.04	0.06	19	2'733	< 1'000	135'929	90	0	50	40	PD	√

[a)] Considered as an immune responder. Linear regression analysis of all 21 patients showed that their age did not correlate with their frequencies of Melan-A specific T cells (R square = 0.002605, P = 0.83).
[b)] Defined as clinically palpable and/or radiologically enlarged
PD: Progressive disease; SD: Stable disease

Supporting Information Figure 1

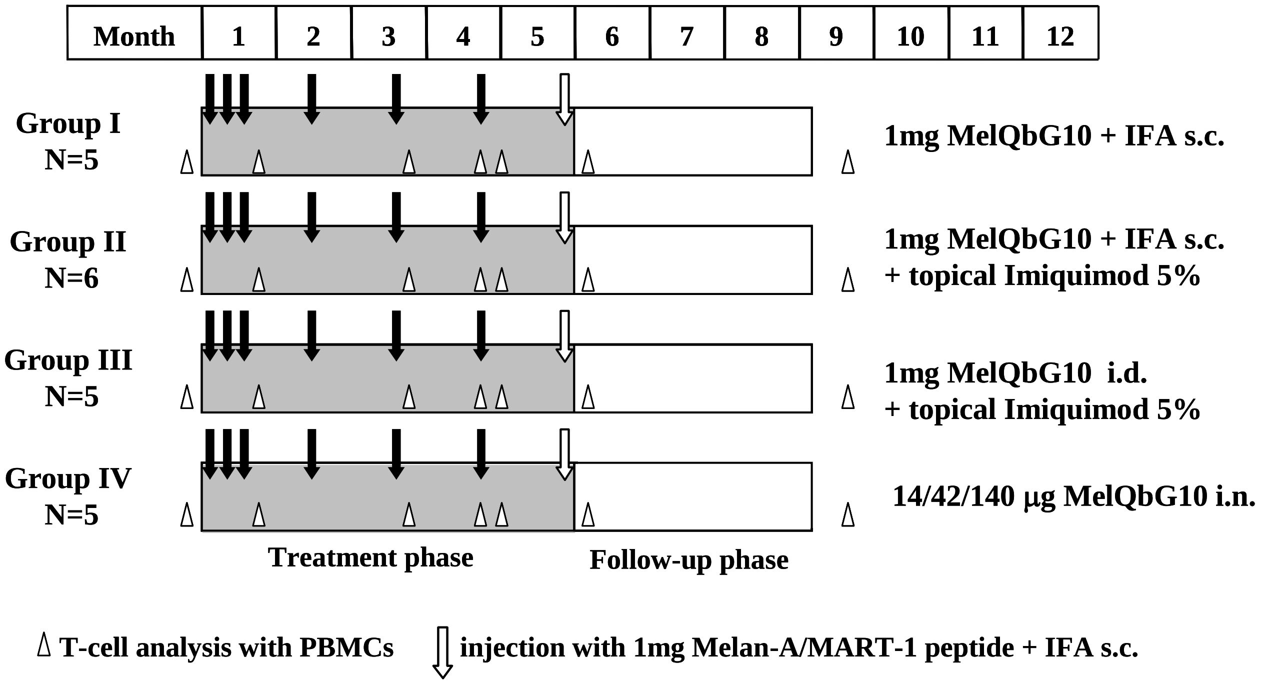

Trial Design. Four groups of patients received vaccinations as indicated on the right side of the figure. For each patient, the total trial duration was approximately 9 months. The screening period took up to 4 weeks. The treatment phase, including the seven injections with MelQbG10, lasted 18 weeks and the follow-up phase ended after 36 weeks. All s.c. and i.d. injections were performed in the upper arm left or right alternatively. Injections with IFA were prepared by mixing equal volumes of MelQbG10 with IFA until the two liquids formed a homogenous emulsion. The intranodal injections were performed always in the same inguinal lymph node region. The lymph node dissected region was omitted for vaccine injections. No immunosuppressive medication was used during the study.

Supporting Information Figure 2

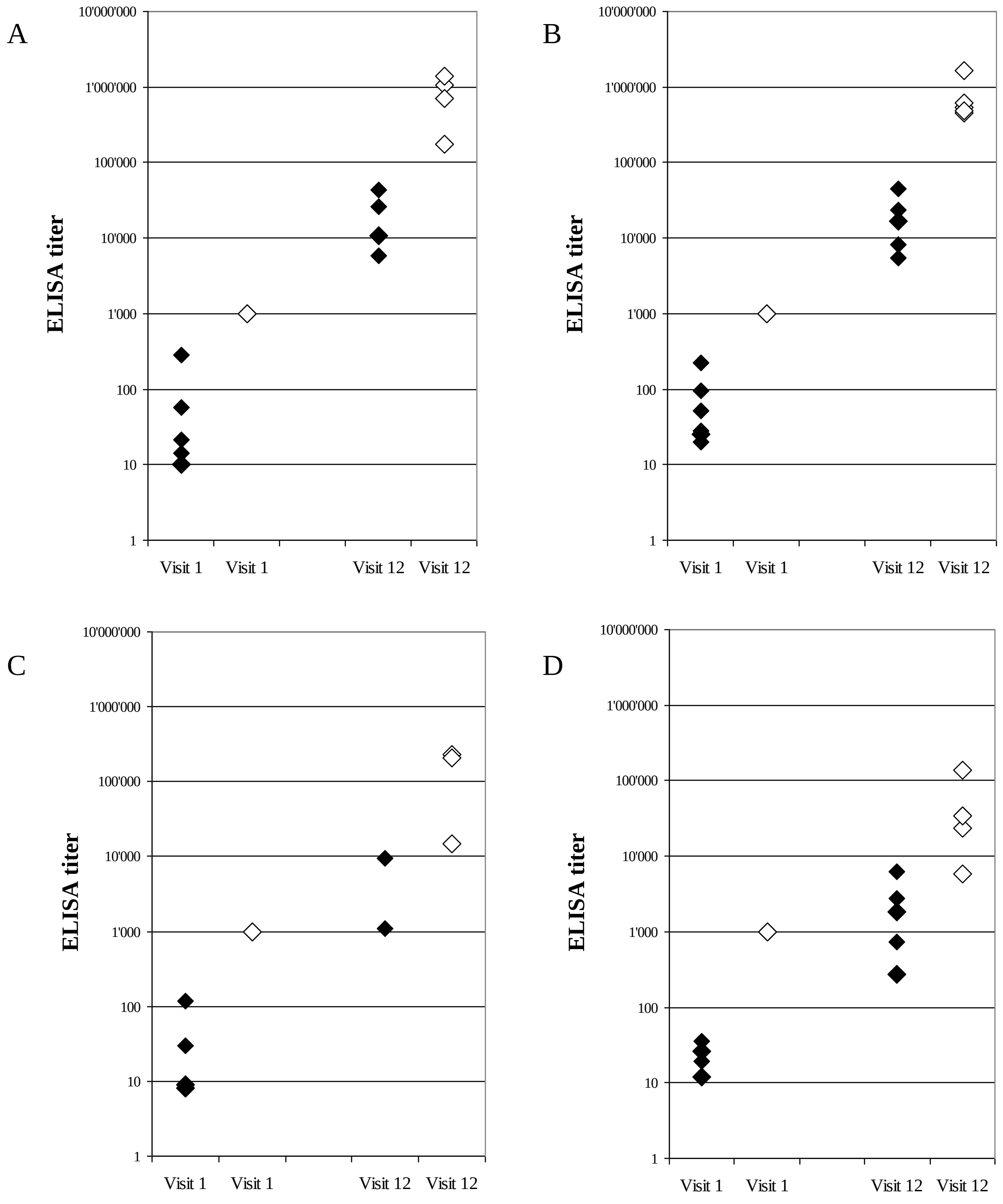

Anti-Melan-A and anti-Qb IgG Antibody responses. Geometric mean titers are expressed as reciprocal serum dilutions giving half-optical density in ELISA. Before vaccination (Visit 1) and at study end (Visit 12) anti-Melan A (♦) and anti-Qb (◊) titers were measured in patients' blood serum samples. **Panel A** shows the titers of group I (1mg MelQbG10 + IFA s.c.), **Panel B** of group II (1mg MelQbG10 + IFA s.c. + Imiquimod 5%), **Panel C** of group III (1mg MelQbG10 i.d.+ Imiquimod 5%), and **Panel D** of group IV (14/42/140mg MelQbG10 i.n.). Mann-Whitney pair wise comparisons were performed. The contrasts of Group I and II vs. Group III and IV were statistically significant for anti-Melan-A and anti-Qb titers (p=0.003 and p=0.001, respectively).

Supporting Information Figure 3

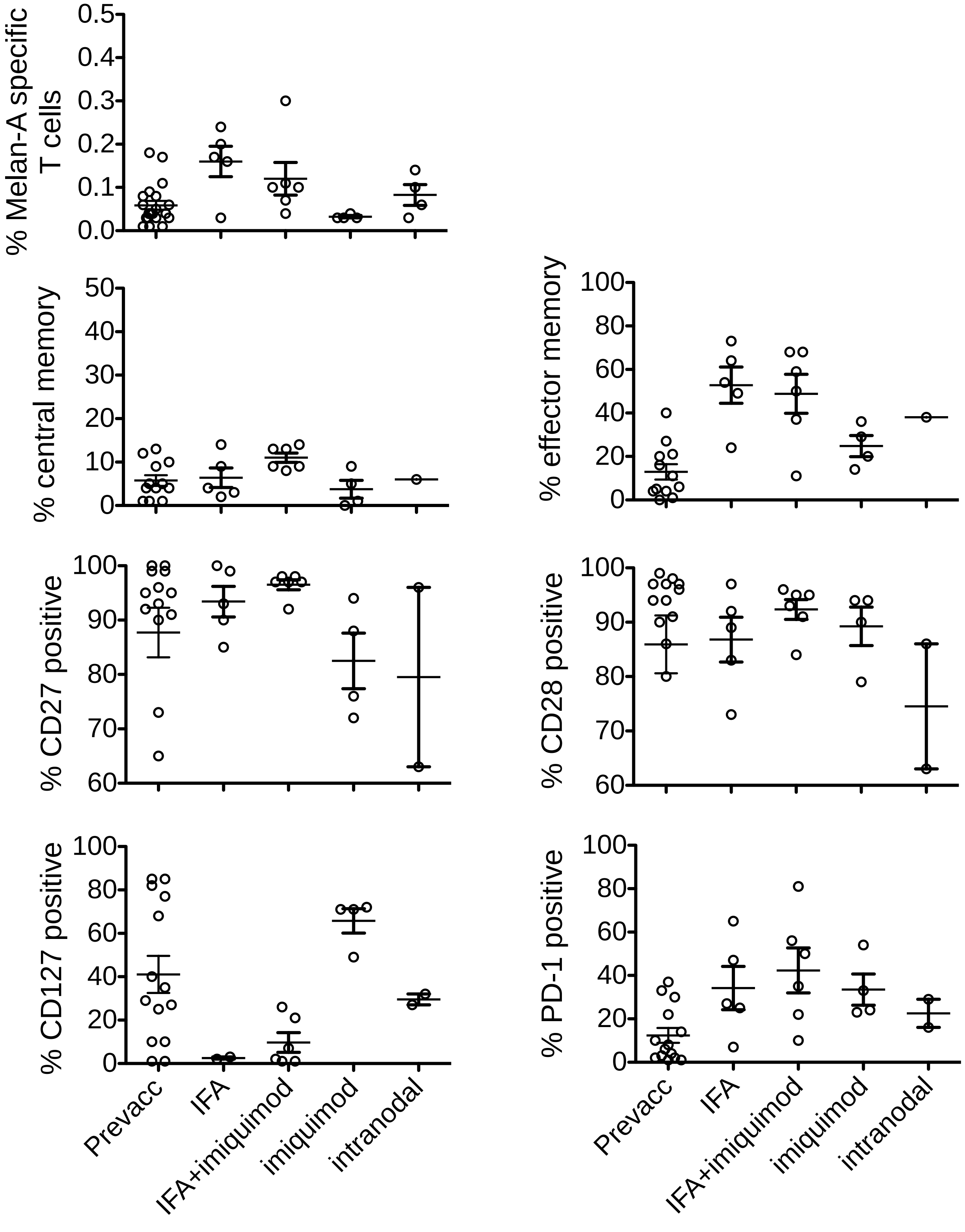

Frequency and memory- / effector-phenotypes of Melan-A specific T cells. Results before vaccination (Prevacc) and of a single time point after vaccination with MelQbG10 (six vaccinations), from the same experiments as those for Figures 2 and 3, shown in a similar fashion. Statistical significances have been determined by the Mann-Whitney test. Where significant, P-values are symbolized with ** or * for <0.01 or 0.01-0.049, respectively.
